# Natural history of neurological improvement following complete (AIS A) thoracic spinal cord injury across three registries to guide acute clinical trial design and interpretation

**DOI:** 10.1038/s41393-019-0299-8

**Published:** 2019-06-10

**Authors:** Alex A. Aimetti, Steven Kirshblum, Armin Curt, Joseph Mobley, Robert G. Grossman, James D. Guest

**Affiliations:** 1 InVivo Therapeutics Corporation, Cambridge, MA USA; 2 Kessler Institute and Rutgers University, West Orange, NJ USA; 3 Spinal Cord Injury Center, Balgrist University Hospital, University of Zurich, Zurich, Switzerland; 4 Department of Neurological Surgery, University of Louisville, Louisville, KY USA; 5 Department of Neurosurgery, Houston Methodist Neurological Institute, Houston, TX USA; 6 Department of Neurosurgery, University of Miami, Miami, FL USA

**Keywords:** Clinical trials, Spinal cord diseases

## Abstract

**Study design:**

Retrospective, longitudinal analysis of motor and sensory outcomes following thoracic (T2–T12) sensorimotor complete spinal cord injury (SCI) in selected patients enrolled into three SCI) registries.

**Objectives:**

To establish a modern-day international benchmark for neurological recovery following traumatic complete thoracic sensorimotor SCI in a population similar to those enrolled in acute clinical trials.

**Setting:**

Affiliates of the North American Clinical Trial Network (NACTN), European Multicenter Study about Spinal Cord Injury (EMSCI), and the Spinal Cord Injury Model Systems (SCIMS).

**Methods:**

Only traumatic thoracic injured patients between 2006 and 2016 meeting commonly used clinical trial inclusion/exclusion criteria such as: age 16–70, T2–T12 neurological level of injury (NLI), ASIA Impairment Scale (AIS) A, non-penetrating injury, acute neurological exam within 7 days of injury, and follow-up neurological exam at least ~ 6 months post injury, were included in this analysis. International Standards for Neurological Classification of Spinal Cord injury outcomes including AIS conversion rate, NLI, and sensory and motor scores/levels were compiled.

**Results:**

A total of 170 patients were included from the three registries: 12 from NACTN, 64 from EMSCI, and 94 from SCIMS. AIS conversion rates at approximately 6 months post injury varied from 16.7% to 23.4% (21.1% weighted average). Improved conversion rates were observed in all registries for low thoracic (T10–T12) injuries when compared with high/mid thoracic (T2–T9) injuries. The NLI was generally stable and lower extremity motor score (LEMS) improvement was uncommon and usually limited to low thoracic injuries only.

**Conclusions:**

This study presents the aggregation of selected multinational natural history recovery data in thoracic AIS A patients from three SCI registries and demonstrates comparable minimal improvement of ISNCSCI-scored motor and sensory function following these injuries, whereas conversions to higher AIS grades occur at a frequency of ~20%. These data inform the development of future clinical trial protocols in this important patient population for the interpretation of the safety and potential clinical benefit of new therapies, and the potential applicability in a multinational setting.

**Sponsorship:**

InVivo Therapeutics.

## Introduction

Traumatic spinal cord injury (SCI) affects ~ 17,000 individuals each year in the United States [1], often resulting in significant impairments of motor, sensory, and autonomic functions as well as substantial financial burden. Safe and effective treatment options to reduce the adverse clinical consequences of the injury are highly needed. To date, multiple therapies intended to neuroprotect or repair the damaged spinal cord have been evaluated in clinical trials [2], yet, none have achieved regulatory approval for use in this patient population. One of the challenges facing clinical development of promising treatments for acute SCI is execution of clinical trials and interpretation of the results. The rare incidence of SCI, paucity of validated biomarkers and further patient segmentation based on inclusion/exclusion criteria leads to substantial challenges for trial enrollment and completion [3, 4]. In light of these issues, early-phase open-label clinical trials are typically conducted to assess both safety and preliminary effectiveness, however interpretability of the results is often difficult. Conventionally designed acute placebo-controlled trials require the enrollment of hundreds of patients and take years to complete [5]. Future acute SCI clinical trial efficiencies are needed to safely and expeditiously advance the clinical development lifecycle of investigational treatments. An example would be the use of adaptive clinical trial designs [6] as proposed by the ADAPT-IT (Adaptive Designs Accelerating Promising Treatments Into Trials) project [7]. In addition, reliable imaging [8] or injury biomarkers from serum are in development [9] to more accurately stratify spinal cord-injured persons.

The importance of *real world data* and its use in regulatory decision making has increased significantly in recent years [10]. In December 2018, the FDA published a frame work for a Real World Evidence Program. https://www.fda.gov/media/120060/download. Real world data can be used to bolster the clinical evidence of the safety and effectiveness of an investigational therapy. These data can come from a variety of sources including electronic medical records, claims, and billing activities, and importantly, patient registries. Several SCI patient data registries exist today that prospectively collect data documenting the natural history of recovery following injury. Three of those registries are the Spinal Cord Injury Model Systems (SCIMS), North American Clinical Trials Network (NACTN), and the European Multicenter Study about Spinal Cord Injury (EMSCI). These registries have been in existence for several decades and collect patient data with a broad geographic reach, including North America and Europe. All three registries collect neurological-based data including outcomes associated with the International Standards for Neurological Classification of Spinal Cord Injury (ISNCSCI) as well as important functional measures including the Spinal Cord Independence Measure (SCIM) and Functional Independence Measure (FIM). These are commonly used outcome measures in acute SCI clinical trials. Data from these registries can be used to understand the natural history of neurological or functional improvement in this patient population. Further, the patient data may be selected to match the most-common clinical trial inclusion criteria. These changes are critical in establishing what a minimal clinically important difference (MCID) should be for any investigational therapy [11].

Previous literature describing the natural history of recovery following acute SCI has been extremely valuable to the field. Reports that focus their analysis on recovery following complete traumatic thoracic injuries are of particular importance, as clinical trials evaluating investigational invasive therapies for acute SCI are typically initiated in this patient population as the risk for neurological deterioration and the functional implication of such loss is lower than at cervical levels. Understanding the spontaneous rate of recovery in this patient population is important when assessing the safety and effectiveness of new therapies [12–14] and in designing new clinical trials. Zariffa et al. [15] reported an American Spinal Injury Association [ASIA] Impairment Scale (AIS) conversion rate of 15.6% at 24 weeks following complete (AIS A) thoracic SCI using data from the EMSCI registry. Similarly, Lee et al. [16] published a 15.5% conversion rate at 1 year following thoracic AIS A spinal cord injury using the SCIMS registry. Furthermore, the Sygen clinical study database, which enrolled 760 people including thoracic complete injuries, has been a rich source of data over the past few decades [17]. Multiple reports have been published summarizing the results including changes in AIS grade, motor score, sensory scores, as well as additional autonomic function outcomes [17–20]. A recent report from NACTN examined the natural history of recovery, including AIS conversion, following thoracic injury and reported a 14.3% conversion rate [21]. Collectively, these reports have helped to set a benchmark in the SCI community around the expected rate of neurological improvement following thoracic AIS A traumatic injuries. A recent summary of previous studies reported that the overall conversion rate noted in thoracic injuries was 30.6% [20]. However, a diversity of studies included in this review did not require an early examination and follow-up varied from 3 to 12 months.

Despite these reports, some previous natural history recovery rates have limited utility for designing and interpreting modern day clinical trials. Previous papers reporting on the natural history of recovery in thoracic AIS A injuries included all-comers entered to their registries in their analysis. However, that breadth is not the case with clinical trial patient selection. Generally, age range is confined, penetrating injuries are excluded, and other filtering criteria are applied in clinical trials in attempts to isolate a more homogenous population that is not at an increased safety risk for participating in a clinical trial. Thus, the rate of recovery in an all-comer population is likely not the same as a more selected clinical trial patient cohort due to the potential presence of greater diversity in prognostic variables. In addition, there has been a major evolution in standard of care with a recommendation toward early surgery [22] and subsequent earlier mobilization. Although the influence that decompressive surgery has on neurological recovery remains incompletely understood, contemporary benchmark data should be limited to a recent time frame and ensure standard of care treatment was performed. Last, the neurological exam to assess SCI patients has also changed over time making it inappropriate to compare modern day trial results with historical data [23, 24]. There is a significant need to provide contemporary benchmark data for thoracic AIS A patients that approximate clinical trial eligibility in order to better interpret clinical data coming out of early phase, open-label studies as well as aid in the design of late-stage clinical studies.

Here we present for the first time a compilation of contemporary, ISNCSCI-based neurological recovery data from three established SCI registries: NACTN, EMSCI, and SCIMS. Patients included in the analysis represent those that best match individuals entered into clinical trials and were treated with modern day standard of care. This effort is aimed at developing a robust comparator group to which data from interventional clinical studies in thoracic AIS A patients can be benchmarked. It is our intention that these findings will guide the development of future clinical trial protocols as well as aid in the interpretation of the safety and potential clinical benefit of new therapies.

## Methods

### Data sources

The selection of subjects for clinical trials is based on defined inclusion and exclusion criteria. The most-commonly applied longitudinal measure is the ISNCSCI [25]. The research question was to determine the proportion of selected subjects with traumatic thoracic SCI that have an initial motor complete AIS A injury and subsequently experience a change in their AIS grade. Only spinal cord levels, T2–T12 (and not L1) were included. To conduct the analysis, three major SCI databases were specifically queried, NACTN, EMSCI, and SCIMS in accordance with their data release policies to provide the requested deidentified data. These registries collectively provide the most comprehensive and robust collection of longitudinal data describing the natural functional recovery of patients following traumatic SCI. Summaries of each organization’s registry are listed below. Each of these registries applies the ISNCSCI exam rigorously requiring that personnel conducting neurological exams are trained to conduct the ISNCSCI [26]. In the time period 2006–2016, the most-substantial update to ISNCSCI was published in 2011 and a revised worksheet in 2013, and an update in 2015. However, the definition of a neurologically complete (AIS A) injury did not change and remained based upon the sacral sparing criteria [27].

#### NACTN

NACTN is an initiative of the Christopher Reeve Paralysis Foundation. Funding to maintain the Registry (NCT00178724) has been obtained from the US Department of Defense. The participating centers have been civilian and military academic neurosurgical hospitals, of which there are currently 12. The registry collects initial clinical status, demographics, detailed medical history, classification of neurological, and bony injury, the type and timing of surgical therapy, adverse events and magnetic resonance imaging data. The Registry seeks to establish the natural history of recovery using standardized and validated measures, to facilitate scholarly research, and to serve as a comparison group in clinical trials [28]. The enrollment of subjects occurs as soon after injury as feasible based on obtaining consent to participate in data collection and follow-up. To be enrolled, consent must be obtained, and the subject must be cognitively capable of undergoing the detailed neurological testing that is employed. The registry currently has data on 938 participants. Those people with SCI who cannot be accessed owing to complex polytrauma, significant head injury, or other altered mental status are not included. Research coordinators who conduct the ISNCSCI and other exams are systematically trained. The data are reviewed frequently for inconsistencies or errors and these are corrected.

#### EMSCI

The aim of the EMSCI project is to establish a multicenter basis for future therapeutic interventions in human SCI. The registry (NCT01571531) was established in 2001 and currently includes over 4500 participants at ~ 18 European centers. EMSCI includes a data quality management system and is ISO 9001 certified demonstrating the organization’s commitment to quality. Participants undergo an acute exam with follow-up assessments performed by trained examiners at 4, 12, 24, and 48 weeks. The examinations consist of a standard set of neurological and functional assessments (www.emsci.org) [29].

#### SCIMS

The SCIMS program was founded in 1970 and during this time, 30 hospitals have served as sites for data collection. More recently, during the 2011–2016 funding cycle, 14 sites in the United States were designated as SCIMS centers. The database was formed in 1975 to collect, manage, and analyze the large amount of data the sites were collecting. Currently, the registry includes approximately 45,000 people with SCI, of whom ~29,000 had one or more follow-up records. Form I data include demographic information and acute care/diagnosis information. Form II data include sociodemographic and outcome data of Form I participants obtained at follow-up [30]. These data are reviewed for inconsistencies or errors and these are corrected.

### Patient inclusion

Patient selection conditions were approximately similar to typical acute SCI clinical trial inclusion/exclusion criteria. In addition, the selection conditions were similar between registries to the extent that the relevant data fields were captured for each data source. Table 1 illustrates the inclusion/exclusion criteria for each registry. Briefly, for each registry patients were included if they provided informed consent, sustained a traumatic SCI between 2006 and 2016, were between the ages of 16 and 70, were classified with a thoracic (T2–T12 neurological level of injury [NLI]), complete (AIS A) injury within 7 days of injury, and had follow-up neurological data beyond approximately 6 months post injury. Six months post injury was selected as an appropriate follow-up duration as this is when neurological recovery generally plateaus and it is the primary endpoint time for many clinical trials [16]. Those persons in the registries not meeting these criteria are not included in this analysis. Additional criteria included patients that underwent acute spinal surgery and excluded patients with penetrating injuries.

**Table 1:** Inclusion/exclusion criteria for patient selection from each registry

	NACTN	EMSCI	SCIMS
Traumatic injuries only	✓	✓	✓
Injured between 2006 and 2016	✓	✓	✓
Baseline ISNCSCI exam (days post injury)	≤ 4	≤ 7	≤ 4
Baseline NLI = T2–T12	✓	✓	✓
AIS A	✓	✓	✓
Age 16–70	✓	✓	✓
Follow-up ISNCSCI exam (months post injury)	5–7	5–6	6–18
Exclude penetrating injuries	✓	< 1%	✓
Spinal surgery required (acute)	✓	> 90%	✓

### Outcome measures

For this analysis, ISNCSCI-based outcome measures are presented including AIS grade conversion, change in NLI, and changes in motor and sensory scores. The ISNCSCI neurological exam is the most-commonly utilized exam to classify spinal cord injuries [23]. In brief, AIS grade is a component of the ISNCSCI exam and remains a widely used outcome measure in SCI clinical trials, particularly those that enroll only thoracic-level injuries. The assessment of complete (AIS A) versus incomplete (AIS B, C, or D) injuries relies solely on the absence or presence, respectively, of sacral sparing [31]. Sacral sparing is measured by sensory testing (light touch and pin-prick) of the well-defined S4–5 dermatome as well as deep anal pressure of the anorectal wall and voluntary anal contraction (VAC). Next, the NLI signifies the most caudal segment of the spinal cord with normal sensory and motor level on both sides of the body. For the majority of thoracic-level SCI patients, the NLI is determined solely on sensory testing of thoracic dermatomes. Negative NLI changes imply rostral deterioration, whereas positive NLI changes signify caudal improvement. Motor testing evaluates 10 myotomes bilaterally with each myotome receiving an ordinal score of 0–5. The maximum total motor score for both upper and lower extremities is 100. It is fairly common for thoracic AIS A patients to have intact upper extremity motor scores of 50 and lower extremity motor scores of 0 unless there are upper extremity fractures or nerve injuries to diminish the motor score or a large zone of partial preservation in the lower extremities to increase the score. At last, sensory testing consists of measuring 28 dermatomes bilaterally using two different stimuli (light touch (LT) and pin-prick (PP)) to assess the dorsal columns and spinothalamic tract.

### Statistical methods

All results are presented using descriptive statistics with no a priori hypothesis testing planned or performed. All summary results are presented for each patient that met criteria from each registry and aggregated where appropriate. Further, as NLI is a known prognostic variable [15, 32], AIS conversion rate and motor scores are presented using an NLI stratification scheme of T2–T5, T6–T9, and T10–T12 patients. The overall weighed average conversion percentage was calculated as NACTN conversion % (12) + EMSCI conversion % (64) + SCIMS conversion % (75)/170.

## Results

### Patient demographics

A total of 170 patients from the three registries were included in this analysis: 12 from NACTN, 64 from EMSCI, and 94 from SCIMS (Table 2). 76.5% (*n* = 130) of the patients were male. The average age at injury ranged from 35.5 years old (SCIMS) to 44.7 years old (NACTN). Patients in the NACTN registry received their baseline ISNCSCI exam at ~16 h post injury on average. It has been reported that acute ( <24 h post injury) ISNCSCI exams are reliable if the patient does not exhibit factors such as closed head injury or serious intoxication, which are typical exclusion criteria for clinical trials [33]. EMSCI patients had their baseline neurological exam conducted at 3.8 days post injury on average. Patient selection from the SCIMS registry required that individuals had a baseline ISNCSCI exam within 4 days of injury and the patients from the registry included in this study had their baseline neurological exam conducted at 1.6 days post injury on average. Of note, however, although the motor exam and AIS was obtained at this early time period, the SCIMS database calculates changes in sensory score as the difference at follow-up from rehabilitation admission, which is ~ 2 weeks post injury in this patient population.

**Table 2:** Demographic information and baseline data for patients included in the analysis from each respective registry

		NACTN	EMSCI	SCIMS
Patients included		12	64	94
Date of injury (%)	2006–2008	33.3	31.3	34.0
	2009–2012	50.0	39.1	34.0
	2013–2016	16.7	29.7	31.9
Age at injury		44.7 ± 11.9	38.7 ± 13.1	35.5 ±14.2
% Male		83.3	78.1	74.5
Time to baseline ISNCSCI exam (days)		0.7 ± 0.9	3.8 ± 2.4	1.6 ±1.3
Baseline neurological level of injury (%)	T2–T5	33.3	26.6	25.5
	T6–T9	50.0	26.6	28.7
	T10–T12	16.7	46.9	45.7

Patients from the EMSCI and SCIMS registries had very similar NLI distributions. NLI is a known predictive factor of recovery with lower level injuries more prone to experience neurological improvement [15, 16, 34]. NACTN had a decreased portion of T10–T12 injuries (16.7%), this should be considered when interpreting summary data. This does not align with the epidemiology of SCI and is likely owing to the small sample size or patterns of practice.

### Follow-up neurological exam

All patients were required to have a follow-up neurological exam beyond approximately 6 months post injury. Each registry has different longitudinal follow-up criteria. Details for when the follow-up ISNCSCI exam occurred for patients from each registry is as follows. Data used for patients within the NACTN registry were collected on average at 184 days (median: 181.5 days) post injury. Similarly, EMSCI patients were evaluated, on average, at 167 days (median: 168 days) post injury. Conversely, the SCIMS registry captures their Year 1 follow-up data at 12 months post injury with a 6-month tolerance. Follow-up data from SCIMS presented here were obtained, on average, at 358 days (median: 361 days) post injury. Although neurological conversion usually occurs early post injury and recovery typically plateaus at ~ 6 months, the difference in follow-up exam timing is important to note when comparing results between registries.

### AIS conversion

**Fig. 1:**
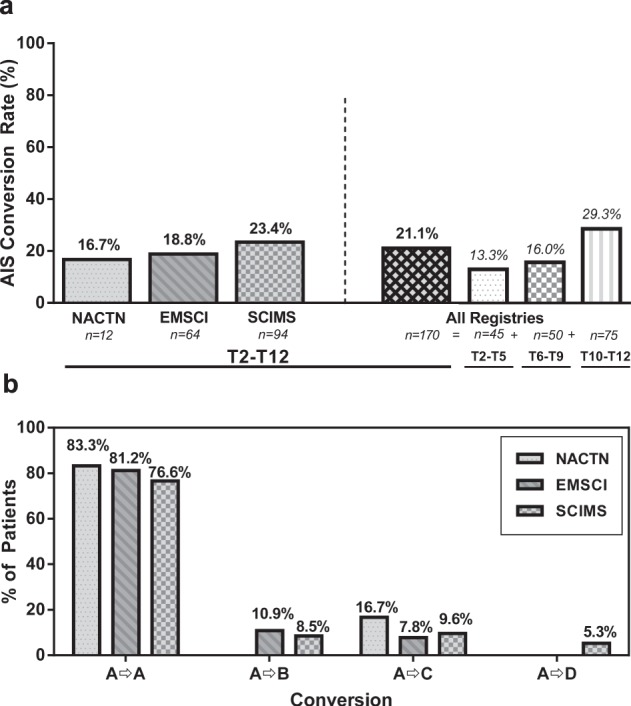
**a** AIS conversion rate for all patients (T2–T12) in each registry as well as a combined weighted average. AIS conversion rate for patients combined from each registry stratified by their baseline NLI (T2–T5, T6–T9, T10–T12). **b** Degree of AIS conversion, if any, to AIS B (A → B), AIS C (A → C), or AIS D (A → D)

AIS conversion rates were approximately similar between the three registries (Figure 1a); 16.7% (2/12), 18.8% (12/64), and 23.4% (22/94) of the patients in the NACTN, EMSCI, and SCIMS registries, respectively, experienced an AIS grade conversion at follow-up visit. The weighted average AIS conversion rate for all registries combined was 21.1% (36/170, 95% CI 15.7–28.0%). The rate of AIS grade improvement was greater with lower level injuries (e.g., T10–T12). In all, 29.3% of these patients experienced an AIS grade conversion compared with 16.0% of T6–T9 patients and 13.3% of T2–T5 patients.

AIS grade improvement rates to either sensory incomplete (AIS B) or motor incomplete (AIS C) were similar between EMSCI and SCIMS (Fig. 1b). The two patients that experienced an AIS grade conversion in the NACTN registry improved to AIS C; one patient had no VAC but a + 1 LEMS and the other patient has no LEMS improvement but had regained VAC. Improvement to AIS D in this patient population is very rare with only five (2.9%) patients, all from the SCIMS registry, with injuries in the T10–T12 region, doing so.

### Change in NLI

**Fig. 2:**
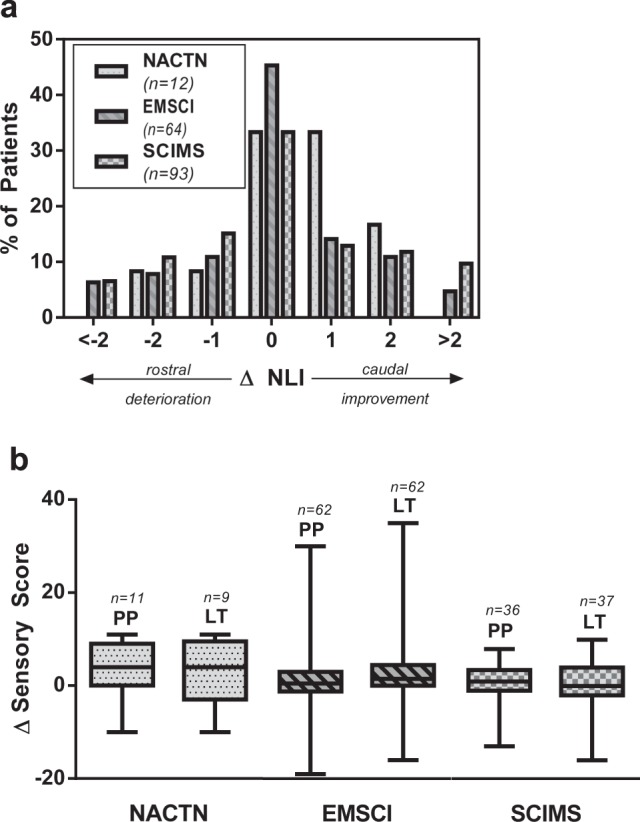
**a** NLI change. Negative integers indicate rostral deterioration and positive integers indicate caudal improvement. Zero indicates no change. **b** Change in sensory scores for both pin-prick (PP) and light touch (LT) for patients in each registry. Box-and-whisker plot indicates min-max and 25th, 50th, and 75th percentiles. Note: the SCIMS database calculates changes in sensory score as the difference at follow-up from rehab admission which is approximately 2 weeks post injury in this patient population

The majority of patients experienced minor changes in NLI (Figure 2a). In all, 33.7% of patients experienced a positive NLI change (i.e., caudal improvement), whereas 28.4% of individuals were documented to have a negative NLI change (i.e., rostral deterioration). Only 5.9% of all patients had an ascent of NLI of more than two levels. Although small variations in thoracic dermatomal levels have little effect on the patients function and QoL, these data are important to understand when evaluating the safety of investigational therapies that may transition to cervical level patients.

### Change in sensory scores

The change in sensory scores (PP and LT) were similar between all three registries (Fig. 2b). The median change in scores was positive for patients in all registries with the exception of the change in LT scores for patients from the SCIMS registry (median ∆LT = 0).

### Change in total motor score

**Fig. 3:**
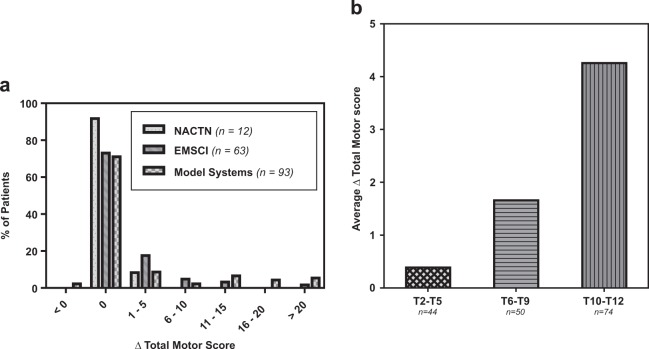
**a** Change in total motor score for patients in each registry. **b** Average change in total motor score for patients combined from each registry stratified by their baseline NLI (T2–T5, T6–T9, T10–T12)

A majority portion of the patients in all three registries experienced no change in total motor score (Fig. 3a). If improvement did occur, it was most likely to be a relatively small change between 1 and 5 motor points. Only 10.6% of the total patients (18/170) experienced a motor score improvement of >10 points with the majority of those patients (14/18) having T10–T12 baseline NLIs. Only two patients, from the SCIMS registry, experienced a motor score loss. These individuals were high level thoracic (T2–T5) injuries and lost upper extremity motor points. Improvement in motor score is correlated to baseline NLI with limited average improvement in T2–T5 injuries (mean: 0.39 points) and increased improvement in T10–T12 injuries (mean: 4.26 points). The median change in total motor score was 0 within all registries and within all NLI strata. Only the 75% percentile within the T10–T12 patients in the EMSCI and SCIMS registries were non-zero with gains of 3.5 and 11 motor points, respectively.

## Discussion

This analysis provides a modern benchmark for expected changes in standardized outcomes after traumatic thoracic complete SCI. Here, we queried three established SCI registries to model selection of clinical trial eligible patients and assess their neurological changes at 6 months (or beyond) post injury. Emphasis was placed on maintaining consistency in the inclusion/exclusion criteria for each registry to maximize the interpretability of cross-registry comparisons. However, this was not fully achieved in all cases owing to variability between registries. For example, all patients included from the NACTN and SCIMS registries had their baseline neurological exam performed within 4 days of injury, whereas patients included from the EMSCI registry had their baseline exam performed within 7 days of injury (median: 4.0 days). Although the reliability of acute ISNCSCI exams has been reported [33], additional work is needed to understand the stability of early neurological exams following traumatic injury. Also, post injury follow-up time was consistent for patients from the NACTN and EMSCI registries (5–7 months), however, SCIMS Form II data collection occurs at 1 year ± 6 months post injury.

AIS grade conversion is the most commonly used clinical endpoint for thoracic SCI trials because this reflects recovery of long tract functions. Improvement in AIS A grade to incomplete injuries, including AIS B, has been correlated to bladder/bowel awareness, decrease in incidence of pressure ulcers [35, 36], and decreased re-hospitalizations [31, 37]. Here, we report a 21.1% pooled AIS conversion rate, which slightly exceeds some of the previous published literature [15, 16, 38]. The higher rates of AIS conversion, particularly in T2–T5, may reflect changes in modern care that emphasize decompressive surgery, support of blood pressure, and very careful transfers to avoid iatrogenic exacerbation. Further, as previously reported, patients with T10–T12 injuries have a more-favorable prognosis for AIS conversion. This information is critically important to recognize in the design of clinical trials and interpretation of the resulting clinical data. The L1 level was not included as injuries at this level may be a mixture of conus and root injury, are generally burst fractures that are often managed conservatively [39], and have a better natural history for recovery [40, 41].

Although small changes in NLI following thoracic injuries lead to little clinical or QoL impact, it is important to accurately document these changes as these data could be useful in assessing a therapeutics risk/benefit. Therefore, there is a need to maintain high inter- and intrarater accuracy and consistency, especially when considering multicenter clinical trials. In complete thoracic injuries, NLI is typically dictated by sensory level solely as motor testing is not currently conducted in this anatomical region. To minimize the subjectivity associated with sensory testing as well as variations in dermatomal mapping [42], it may be useful to physically mark the observed sensory levels and photograph them in order to be more certain of NLI changes [43]. At last, the absence of motor testing from T1 to L1 represents a gap in classification and measures of trunk functions [44] need further development by the SCI community in order to understand the clinical meaningfulness of level-by-level improvement in the thoracic patients.

Motor improvement is uncommon following thoracic AIS A injuries that precludes its use as a preferred outcome measure in this patient population in the absence of a therapeutic with a large effect size. Although motor improvement is a desired clinical outcome, it is not of the highest priority for paraplegics [45], further supporting the use of alternative primary outcome measures.

These data presented herein, collectively, can be used to help interpret the safety and preliminary effectiveness of novel therapeutics in early-stage clinical trials. Further, this information can help guide the design of follow-on trials. Based on a 21.1% standard of care AIS conversion rate including all thoracic (T2–T12) levels, a randomized controlled clinical trial designed to show a 20% AIS conversion rate difference, which one prior study published as a potential MCID [4], would have to enroll approximately 80 subjects in each group (*α* = 0.05, *β* = 0.80). Based on previous clinical trial enrollment rates in this patient population, a trial of that size would be difficult to execute, and alternative trial designs may be needed to advance treatment options in this area of significant unmet medical need. However, the good comparability of changes in the thoracic AIS A patients across the different SCI networks based in American and European countries indicates that multinational trials in this patient population should be feasible and reliable. The MCID after SCI remains without clear definition, especially for thoracic injuries where segmental recovery has less impact [11]. Conversion to AIS B or higher has been linked to less frequent and lengthy hospitalizations, which could be perceived to increase quality of life [37]. In a multiple Logit model regression, it was found that odds ratios for important aspects of daily life differed between AIS A and AIS B subjects, for example AIS B subjects less frequently had indwelling catheters at discharge from rehabilitation and at one year follow-up [31].

This analysis comes with various limitations. First, patients included in this analysis were required to have a follow-up neurological exam, therefore those that were lost to follow-up were excluded. This introduces bias into the natural history data presented here although the exact impact is unknown. Also, this is different from a typical clinical trial where all efficacy analyses are conducted on the intent to treat group, which includes all patients randomized even if they subsequently withdraw or are lost to follow-up. The withdrawal or lost to follow-up rates from the registries are not reported here but are likely higher than what one would expect in a clinical trial. Also, it was not possible to maintain fully consistent inclusion/exclusion criteria for patients across registries owing to the different data fields each organization collects. To identify a more homogenous patient population, similar to clinical trials, it is recommended that the organizations collaborate to ensure critical demographic information, mechanism of injury, and outcomes data are collected at consistent time points to allow for better registry to registry comparison and compilation. We see this work as an initial product of collaboration across the three registries. Data sharing is complex between established registries and here only aggregate data from SCIMS and EMSCI was provided. Some of the barriers have been articulated [46] and a further effort is underway to facilitate inter-registry data sharing known as the International Spinal Data Network.

In conclusion, this study presents the aggregation of recovery data in thoracic AIS A patients from three multinational SCI registries and demonstrates comparable minimal improvement of motor and sensory function following these injuries, whereas conversions to higher AIS grades occur at a frequency of ~20%. These data inform the development of future clinical trial protocols in this patient population for the interpretation of the safety and potential clinical benefit of new therapies, and the potential applicability in a multinational setting. Future studies may refine this conversion rate, but it is consistent with numerous improvements in clinical practice before and during the study period.

## Data Availability

All data presented here were obtained, stored, and analyzed by the respective registry organizations.
